# Increased Length of Hospital Stay after Endovascular Abdominal Aortic Aneurysm Repair: Role of Pulmonary Complications

**DOI:** 10.7759/cureus.4986

**Published:** 2019-06-24

**Authors:** Yang Yang, Erik Lehman, Faisal Aziz

**Affiliations:** 1 Vascular Surgery, Drexel University College of Medicine, Philadelphia, USA; 2 Surgery, Penn State College of Medicine, Penn State Milton S. Hershey Medical Center, Hershey, USA; 3 Cardiac / Thoracic / Vascular Surgery, Penn State College of Medicine, Penn State Milton S. Hershey Medical Center, Hershey, USA

**Keywords:** evar, respiratory failure, length of stay, aortic aneurysm

## Abstract

Objectives

The average hospital length of stay plays a significant role in healthcare costs, and is also used as a metric of hospital efficiency. An advantage of endovascular abdominal aortic aneurysm repair (EVAR) is the shorter postoperative time period after the surgery. The purpose of this study is to review the factors associated with increased length of stay after EVAR.

Methods

The records from American College of Surgeons National Quality Improvement Program (ACS-NSQIP) database in 2013 were obtained using Procedure Participant User File. Pre-, intra-, and post-operative factors were assessed of patients undergoing EVAR in 2013. Multivariable logistic regression analysis was used to identify independent variables for a hospital length of stay of at least seven days.

Results

A total of 1,991 patients (18.7% female, 81.3% males) underwent EVAR in 2013. Among these patients, 223 (11.2%) had a hospital stay greater than seven days. Variables significantly associated with length of stay in a multivariable model included: total operation time greater than 180 minutes (vs. less than 90 minutes, OR 1.88, CI 1.03-3.41, p = 0.039), postoperative, and intraoperative transfusions (OR 2.60, CI 1.66-4.08, p < 0.001), return to operating room (OR 2.88, CI 1.55-5.38, p < 0.001), rupture indication for surgery (OR 5.59, CI 3.18-9.83, p < 0.001), myocardial infarction (OR 5.85, CI 2.22-15.43, p < 0.001), preoperative transfusion (OR 13.05, CI 4.26-39.99, p < 0.001), and on ventilator greater than 48 hours (OR 49.65, CI 10.72-230.07, p < 0.001).

Conclusions

Multiple factors affect length of hospital stay in patients who have undergone EVAR. Patients with postoperative respiratory failure after EVAR have a significantly higher risk for longer hospital stays.

## Introduction

In the United States health care system, there has been an emphasis on providing the best patient care while simultaneously maximizing hospital efficiency and expenditures. One factor that is commonly analyzed is length of stay (LOS), which is used as a proxy for resource utilization and healthcare delivery cost [1-3]. Previous studies have shown that decreased LOS results in significant cost savings [3-5]. By understanding factors that play a role in LOS, hospital administration and leaders may be able to improve unnecessarily long hospital stays without negatively impacting patient outcomes.

Abdominal aortic aneurysms (AAA) with large diameter aneurysms are known to have an increased risk of rupture, and in turn, mortality. This disease process can be treated with either open AAA repair or endovascular abdominal aortic aneurysm repair (EVAR). EVAR is quickly becoming the preferred treatment for patients with AAA, due to its shorter postoperative LOS compared to open repair.

The aim of our study was to examine predictors for a prolonged LOS in patients who have undergone EVAR in order to identify potential modifiable risk factors and areas to improve healthcare delivery. We hypothesize that a number of preoperative, intraoperative, and postoperative variables could be identified and used as targets to reduce LOS in patients undergoing EVAR.

## Materials and methods

Data set

The American College of Surgeons-National Surgical Quality Improvement Program (ACS-NSQIP) is a national outcomes-based, surgical data set comprised of data from over 600 hospitals across the US [6]. It does not identify hospitals, healthcare providers, or patients, thus obviating the need for Institutional Review Board approval or patients’ consent. Patient demographics, pre-operative, intra-operative, and post-operative variables are recorded and maintained by trained clinical nurses at all the participating sites. A systemic sampling method is used to ensure adequate representation of all surgical operations. Outcomes are recorded for 30 days and have been shown to be highly reliable with less than 1.5% variable disagreements in annual audits [7]. The data set is maintained by the ACS and is compliant with the Health Insurance Portability and Accountability Act (HIPAA). Multiple publications based on analyses of this database have been published in literature.

Patients

ACS started using specific Procedure Targeted Participant User Files in year 2011 for certain vascular surgery and colorectal surgical operations. The procedure targeted file for patients who underwent EVAR in year 2013 was utilized. Using unique case identification numbers, this file was merged to the main ACS-NSQIP adult participant use data file for year 2013. Methods used to extract data from this data set have been described in previously published literature [7-11].

Outcomes

The primary outcome was increased length of stay, defined as total hospital stay greater than seven days. Basic demographic data was analyzed including age, gender, race, age range, and body mass index (BMI) range. The complete list of pre-operative, intra-operative, and post-operatives is included in Table 1 and Table 2.

Statistical analysis

All variables were initially summarized with frequencies and percentages or means, medians, and standard deviations. Level of statistical significance was set at p = 0.05. Logistic regression was used to determine any bivariate associations of independent variables with length of stay greater than seven days. Odds ratios were used to quantify the magnitude and direction of any significant associations. The independent variables that were significant in the initial bivariate analysis were then used in a process of stepwise selection to find the group of variables collectively that were most significantly associated with length of stay greater than seven days in a multivariable logistic regression model. Stringent entry and stay criteria of p < 0.05 were used for the stepwise process of variable selection to determine the best multivariable logistic regression model that included the factors most significantly associated with increased length of stay. Prior to any modeling selection, the pool of potential predictor variables were tested for multicollinearity using variance inflation factor (VIF) statistics, and those with VIF statistics greater than four were excluded from consideration. Forward, backward, and best subsets methods of variable selection were also employed to check for other potential models, and all four approaches resulted in a similar reduced model. The fit of the final model was assessed using the Pearson, Deviance, and Hosmer and Lemeshow goodness-of-fit tests. Predicted probabilities for patient characteristics were generated from an equation incorporating the parameter estimates from the model. All analyses were performed using SAS software version 9.4 (SAS Institute, Cary, NC).

## Results

Demographics and pre-operative co-morbidities

A total of 1,991 patients (18.7% female, 81.3% males) underwent EVAR in 2013. Among these patients, 223 (11.2%) had a hospital stay greater than seven days.

Comparing variables between length of stay greater than seven days and less than seven days

Patients were divided into two groups: length of stay greater than seven days (N = 223) and length of stay less than seven days (N = 1,768). The following factors were found to be significantly associated with longer length of stay in bivariate analysis using logistic regression: Black race, dyspnea at rest, dependent functional health status, ventilator dependent, severe chronic obstructive pulmonary disease (COPD), congestive heart failure (CHF), open wound or infection, more than 10% loss of body weight, pre-operative blood transfusion, emergency case, American Society of Anesthesiologists (ASA) classification 4-5: life threatening or moribund, rupture indication for surgery, transferred, access vessels, total operation time greater than 180, lower extremity revascularization, ischemic colitis, lower extremity ischemia, discharge destination, pneumonia, unplanned intubation, ventilator dependency greater than 48 hours, renal insufficiency, acute renal failure, urinary tract infection, stroke with neurological deficits, cardiac arrest requiring cardiopulmonary resuscitation (CPR), myocardial infarction, perioperative transfusions, deep vein thrombosis (DVT), sepsis, septic shock, return to operating room, days from admission to operation greater than one (Table 1).

**Table 1:** Bivariate analysis for variables associated with elective surgery. BMI: Body mass index; HTN: Hypertension; COPD: Chronic obstructive pulmonary disease; ASA: American Society of Anesthesiologists; EVAR: Endovascular abdominal aortic aneurysm repair; SSI: Surgical site infection; CVA: Cerebrovascular accident; CPR: Cardiopulmonary resuscitation; DVT: Deep vein thrombosis; OR: Operating room.

Variable	Total	Length of stay ≥ 7 days (N = 223)	Length of stay < 7 days (N = 1,768)	OR (95% CI)	P-value
Preoperative Variables					
Age (years)					
<60	113 (5.7)	18 (15.9)	95 (84.1)	Reference	0.004
60-69	487 (24.5)	43 (8.8)	444 (91.2)	0.51 (0.28-0.93)	
70-79	796 (40.0)	76 (9.6)	720 (90.4)	0.56 (0.32-0.97)	
≥80	595 (29.9)	86 (14.5)	509 (85.5)	0.89 (0.51-1.55)	
Sex (gender)					
Female	373 (18.7)	55 (14.8)	318 (85.2)	1.49 (1.08-2.07)	0.017
Male	1618 (81.3)	168 (10.4)	1450 (89.6)	Reference	
Race					
Non-Hispanic White	18.24 (91.6)	191 (10.5)	1633 (89.5)	Reference	0.003
Non-Hispanic Black	121 (6.1)	24 (19.8)	97 (80.2)	2.12 (1.32-3.39)	
Hispanic	46 (2.3)	8 (17.4)	38 (82.6)	1.80 (0.83-3.92)	
BMI					
<25 (normal)	515 (26.3)	80 (15.5)	435 (84.5)	Reference	<0.001
25-30 (overweight)	769 (39.3)	68 (8.8)	701 (91.2)	0.53 (0.37-0.75)	
≥30 (obese)	673 (34.4)	65 (9.7)	608 (90.3)	0.58 (0.41-0.83)	
Inpatient/outpatient					
Inpatient	1976 (99.2)	223 (11.3)	1753 (88.7)	Reference	0.980
Outpatient	15 (0.8)	0 (0.0)	15 (100.0)	<0.001 (<0.001->999.99)	
Transferred					
No	1790 (89.9)	136 (7.6)	1654 (92.4)	Reference	<0.001
Yes	201 (10.1)	87 (43.3)	114 (56.7)	9.28 (6.68-12.90)	
Days from admission to operation ≥1					
No	1717 (86.2)	117 (6.8)	1600 (93.2)	Reference	<0.001
Yes	274 (13.8)	106 (38.7)	168 (61.3)	8.63 (6.35-11.73)	
Infrarenal proximal aneurysm extent					
No	1684 (90.0)	30 (16.0)	158 (84.0)	Reference	0.031
Yes	188 (10.0)	180 (10.7)	1504 (89.3)	0.63 (0.41-0.96)	
Distal extent					
Aortic	778 (47.2)	81 (10.4)	697 (89.6)	Reference	0.837
Common iliac	636 (38.6)	68 (10.7)	568 (89.3)	1.03 (0.73-1.45)	
External iliac	97 (5.9)	13 (13.4)	84 (86.6)	1.33 (0.71-2.50)	
Internal iliac	138 (8.4)	14 (10.1)	124 (89.9)	0.97 (0.53-1.77)	
Clean wound					
No	25 (1.3)	2 (92.0)	23 (8.0)	Reference	0.613
Yes	1966 (98.7)	221 (11.2)	1745 (88.8)	1.46 (0.34-6.21)	
Open wound/wound infection					
No	1966 (98.7)	210 (10.7)	1756 (89.3)	Reference	<0.001
Yes	25 (1.3)	13 (52.0)	12 (48.0)	9.06 (4.08-20.11)	
Steroid use for chronic condition					
No	1897 (95.3)	207 (10.9)	1690 (89.1)	Reference	0.070
Yes	94 (4.7)	16 (17.0)	78 (83.0)	1.68 (0.96-2.92)	
>10% loss body weight in last 6 months					
No	1970 (99.0)	214 (10.9)	1756 (89.1)	Reference	<0.001
Yes	21 (1.0)	9 (42.9)	12 (57.1)	6.15 (2.56-14.78)	
Bleeding disorders					
No	1720 (86.4)	186 (10.8)	1534 (89.2)	Reference	0.169
Yes	271 (13.6)	37 (13.6)	234 (86.4)	1.30 (0.89-1.91)	
Pre-op transfusion					
No	1951 (98.0)	194 (9.9)	1757 (90.1)	Reference	<0.001
Yes	40 (2.0)	29 (72.5)	11 (27.5)	23.87 (11.74-48.54)	
Prior abdominal aortic surgery					
No	1286 (71.0)	142 (11.0)	1144 (89.0)	Reference	0.909
Yes	525 (29.0)	57 (10.9)	468 (89.1)	0.98 (0.71-1.36)	
HTN requiring medication					
No	384 (19.3)	46 (12.0)	338 (88.0)	Reference	0.590
Yes	1607 (80.7)	177 (11.0)	1430 (89.0)	0.91 (0.64-1.29)	
Diabetes with oral agents or insulin					
No	1681 (84.4)	186 (11.1)	1495 (88.9)	Reference	0.857
Insulin	69 (3.5)	9 (13.0)	60 (87.0)	1.21 (0.59-2.47)	
Non-insulin	241 (12.1)	28 (11.6)	213 (88.4)	1.06 (0.69-1.61)	
Current smoker within one year					
No	1370 (68.8)	145 (10.6)	1225 (89.4)	Reference	0.196
Yes	621 (31.2)	78 (12.6)	543 (87.4)	1.21 (0.91-1.63)	
Dyspnea					
No	1577 (79.2)	166 (10.5)	1411 (89.5)	Reference	<0.001
Moderate exertion	378 (19.0)	45 (11.9)	333 (88.1)	1.15 (0.81-1.63)	
At rest	36 (1.8)	12 (33.3)	24 (66.7)	4.25 (2.09-8.66)	
Ventilator dependent					
No	1973 (99.1)	212 (10.7)	1761 (89.3)	Reference	<0.001
Yes	18 (0.9)	11 (61.1)	7 (38.9)	13.05 (5.01-34.03)	
History of severe COPD					
No	1638 (82.3)	157 (9.6)	1481 (90.4)	Reference	<0.001
Yes	353 (17.7)	66 (18.7)	287 (81.3)	2.17 (1.59-2.97)	
Congestive heart failure in 30 days prior to surgery					
No	1952 (98.0)	205 (10.5)	1747 (89.5)	Reference	<0.001
Yes	39 (2.0)	18 (46.2)	21 (53.8)	7.31 (3.83-13.94)	
Currently on dialysis (pre-operative)					
No	1967 (98.8)	218 (11.1)	1749 (88.9)	Reference	0.141
Yes	24 (1.2)	5 (20.8)	19 (79.2)	2.11 (0.78-5.71)	
Ischemic colitis					
No	1969 (98.9)	209 (10.6)	1760 (89.4)	Reference	<0.001
Yes	22 (1.1)	14 (63.6)	8 (36.4)	14.74 (6.11-35.54)	
Dependent functional health status prior to surgery					
No	1915 (96.9)	199 (10.4)	1716 (89.6)	Reference	<0.001
Yes	61 (3.1)	19 (31.1)	42 (68.9)	3.90 (2.23-6.84)	
Disseminated cancer					
No	1982 (99.5)	220 (11.1)	1762 (88.9)	Reference	0.051
Yes	9 (0.5)	3 (33.3)	6 (66.7)	4.01 (1.00-16.13)	
Rupture indication for surgery					
No	1819 (93.0)	153 (8.4)	1666 (91.6)	Reference	<0.001
Yes	137 (7.0)	67 (48.9)	70 (51.1)	10.42 (7.17-15.14)	
Rupture of aneurysm					
No	1984 (99.6)	220 (11.1)	1764 (88.9)	Reference	0.019
Yes	7 (0.4)	3 (42.9)	4 (57.1)	6.01 (1.34-27.05)	
Emergency case					
No complication	1801 (90.5)	143 (7.9)	1658 (92.1)	Reference	<0.001
Emergency case	190 (9.5)	80 (42.1)	110 (57.9)	8.43 (6.03-11.79)	
Intraoperative Variables					
Main body device					
Gore Excluder	626 (32.0)	75 (12.0)	551 (88.0)	Reference	0.121
Cook	461 (23.6)	63 (13.7)	398 (86.3)	0.86 (0.60-1.23)	
Medtronic	581 (29.7)	55 (9.5)	526 (90.5)	1.30 (0.90-1.88)	
Other	287 (14.7)	27 (9.4)	260 (90.6)	1.31 (0.82-2.08)	
Iliac branched device					
No	1708 (85.8)	192 (11.2)	1516 (88.8)	Reference	0.888
Yes	283 (14.2)	31 (10.9)	252 (0.65-1.45)	0.97 (0.65-1.45)	
Aortic bare metal stent					
No	1946 (97.7)	219 (11.2)	1727 (88.8)	Reference	0.620
Yes	45 (2.3)	4 (8.9)	41 (91.1)	0.77 (0.27-2.17)	
Iliac bare metal stent					
No	1922 (96.5)	215 (11.2)	1707 (88.8)	Reference	0.915
Yes	69 (3.5)	8 (11.6)	61 (88.4)	1.04 (0.49-2.21)	
General anesthesia					
No	190 (9.5)	22 (11.6)	168 (88.4)	Reference	0.860
Yes	1801 (90.5)	201 (11.2)	1600 (88.8)	0.96 (0.60-1.53)	
ASA classification					
1-2 No/mild disturbance	98 (4.9)	2 (2.0)	96 (98.0)	Reference	<0.001
3 Severe disturbance	1282 (64.4)	80 (6.2)	1202 (93.8)	3.19 (0.77-13.18)	
4-5 Life threatening/moribund	609 (30.6)	141 (23.2)	468 (76.8)	14.45 (3.52-59.35)	
Vascular surgical specialty					
No	54 (2.7)	3 (5.6)	51 (94.4)	Reference	0.193
Yes	1937 (97.3)	220 (11.4)	1717 (88.6)	2.18 (0.67-7.04)	
EVAR access					
Bilateral groin cutdown	1212 (61.1)	142 (11.7)	1070 (88.3)	Reference	0.037
Attempted percutaneous access converted to open cutdown	20 (1.0)	5 (25.0)	15 (75.0)	2.51 (0.90-7.02)	
One groin cutdown	198 (10.0)	27 (13.6)	171 (86.4)	1.19 (0.77-1.85)	
Percutaneous bilateral	553 (27.9)	48 (8.7)	505 (91.3)	0.72 (0.51-1.01)	
Total operation time					
≤90	336 (16.9)	27 (8.0)	309 (92.0)	Reference	<0.001
91-120	497 (25.0)	26 (5.2)	471 (94.8)	0.63 (0.36-1.10)	
121-180	687 (34.5)	75 (10.9)	612 (89.1)	1.40 (0.89-2.22)	
≥180	471 (23.7)	95 (20.2)	376 (79.8)	2.89 (1.84-4.55)	
Acute conversion to open procedure					
No	1968 (99.3)	219 (11.1)	1749 (88.9)	Reference	0.036
Yes	13 (0.7)	4 (30.8)	9 (69.2)	3.55 (1.08-11.63)	
Access vessels (conduit, repair)					
No	1853 (93.1)	195 (10.5)	1658 (89.5)	Reference	<0.001
Yes	138 (6.9)	28 (20.3)	110 (79.7)	2.16 (1.39-3.36)	
Renal stent					
No	1831 (92.0)	194 (10.6)	1637 (89.4)	Reference	0.004
Yes	160 (8.0)	29 (18.1)	131 (81.9)	1.87 (1.22-2.87)	
Hypogastric embolization					
No	1853 (93.1)	201 (10.8)	1652 (89.2)	Reference	0.069
Yes	138 (6.9)	22 (15.9)	116 (84.1)	1.56 (0.97-2.52)	
Hypogastric revascularization					
No	1902 (95.5)	210 (11.0)	1692 (89.0)	Reference	0.298
Yes	89 (4.5)	13 (14.6)	76 (85.4)	1.38 (0.75-2.53)	
Lower extremity revascularization					
No	1907 (95.8)	201 (10.5)	1706 (89.5)	Reference	<0.001
Yes	84 (4.2)	22 (26.2)	62 (73.8)	3.01 (1.81-5.01)	
Postoperative variables					
Discharge destination					
Home	1779 (89.4)	108 (6.1)	1671 (93.9)	Reference	<0.001
Expired	42 (2.1)	17 (40.5)	25 (59.5)	10.52 (5.51-20.08)	
Other	169 (8.5)	97 (57.4)	72 (42.6)	20.84 (14.51-29.92)	
Superficial surgical site occurrence					
No complication	1965 (98.7)	217 (11.0)	1748 (89.0)	Reference	0.061
Superficial incisional SSI	26 (1.3)	6 (23.1)	20 (76.9)	2.42 (0.96-6.08)	
Deep incisional SSI					
No complication	1982 (99.5)	222 (11.2)	1760 (88.8)	Reference	0.993
Deep incisional SSI	9 (0.5)	1 (11.1)	8 (88.9)	0.99 (0.12-7.96)	
Pneumonia					
No complication	1966 (98.7)	202 (10.3)	1764 (89.7)	Reference	<0.001
Pneumonia	25 (1.3)	21 (84.0)	4 (16.0)	45.83 (15.58-134.81)	
Unplanned intubation					
No complication	1950 (97.9)	201 (10.3)	1749 (89.7)	Reference	<0.001
Unplanned intubation	41 (2.1)	22 (53.7)	19 (46.3)	10.01 (5.36-18.94)	
Ventilator >48 hours					
No complication	1949 (97.9)	187 (9.6)	1762 (90.4)	Reference	<0.001
On ventilator greater than 48 hrs	42 (2.1)	36 (85.7)	6 (14.3)	56.54 (23.51-135.93)	
Urinary tract infection					
No complication	1965 (98.7)	215 (10.9)	1750 (89.1)	Reference	0.003
Urinary tract infection	26 (1.3)	8 (30.8)	18 (69.2)	3.62 (1.56-8.42)	
Renal insufficiency					
No complication	1978 (99.4)	215 (10.9)	1763 (89.1)	Reference	<0.001
Progressive renal insufficiency	13 (0.6)	8 (61.5)	5 (38.5)	13.12 (4.25-40.46)	
Acute renal failure					
No complication	1973 (99.1)	209 (10.6)	1764 (89.4)	Reference	<0.001
Acute renal failure	18 (0.9)	14 (77.8)	4 (22.2)	29.50 (9.63-90.42)	
CVA/stroke with neurological deficit					
No complication	1982 (99.5)	217 (11.0)	1765 (89.0)	Reference	<0.001
Stroke/CVA	9 (0.5)	6 (66.7)	3 (33.3)	16.26 (4.04-65.46)	
Cardiac arrest requiring CPR					
No complication	1975 (99.2)	217 (11.0)	1758 (89.0)	Reference	0.002
Cardiac arrest requiring CPR	16 (0.8)	6 (37.5)	10 (62.5)	4.86 (1.75-13.51)	
Myocardial infarction					
No complication	1960 (98.4)	208 (10.6)	1752 (89.4)	Reference	<0.001
Myocardial infarction	31 (1.6)	15 (48.4)	16 (51.6)	7.89 (3.85-16.20)	
DVT/thrombophlebitis					
No complication	1973 (99.1)	210 (10.6)	1763 (89.4)	Reference	<0.001
DVT requiring therapy	18 (0.9)	13 (72.2)	5 (27.8)	21.82 (7.70-61.82)	
Sepsis					
No complication	1968 (98.8)	208 (10.6)	1760 (89.4)	Reference	<0.001
Sepsis	23 (1.2)	15 (65.2)	8 (34.8)	15.87 (6.65-37.87)	
Septic shock					
No complication	1974 (99.2)	210 (10.6)	1764 (89.4)	Reference	<0.001
Septic shock	17 (0.8)	13 (76.5)	4 (23.5)	27.30 (8.82-84.49)	
Lower extremity ischemia					
No	1962 (98.5)	215 (11.0)	1747 (89.0)	Reference	0.007
Yes	29 (1.5)	8 (27.6)	21 (72.4)	3.10 (1.36-7.08)	
Return to OR					
No	1898 (95.3)	185 (9.8)	1713 (90.2)	Reference	<0.001
Yes	93 (4.7)	38 (40.9)	55 (59.1)	6.40 (4.12-9.94)	

Multivariable analysis

The following factors were found to have significant associations with longer hospital stay: total operation time greater than 180 minutes (vs. less than 90 minutes, OR 2.02, CI 1.03-3.41, p = 0.039), postoperative and intraoperative transfusions (OR 2.60, CI 1.66-4.08, p < 0.001), return to operating room (OR 2.88, CI 1.55-5.38, p < 0.001), rupture indication for surgery (OR 5.59, CI 3.18-9.83, p < 0.001), myocardial infarction (OR 5.85, CI 2.22-15.43, p < 0.001), preoperative transfusion (OR 13.05, CI 4.26-39.99, p < 0.001), and time on ventilator greater than 48 hours (OR 49.65, CI 10.72-230.07, p < 0.001) (Table 2).

**Table 2:** Multivariable analysis for variables associated with increased length of stay. OR: Operating room; AAA: Abdominal aortic aneurysm.

Variable	OR	95% CI	P-value
Total operation time: >180 vs ≤90	1.88	1.03–3.41	0.039
Post-operative/Intra-operative transfusions	2.60	1.66–4.08	<0.001
Return to OR	2.88	1.55–5.38	<0.001
Ruptured AAA	5.59	3.18–9.83	<0.001
Myocardial infarction	5.85	2.22–15.43	<0.001
Pre-operative transfusion	13.05	4.26–39.99	<0.001
Time on ventilator greater than 48 hours	49.65	10.72–230.07	<0.001

Predicted probability of increased length of stay

The probability of an increased length of stay was calculated for all of the factors to be significant in the multivariable analysis. The probability of an increased length of stay was 8.0% for patients who had operations in the fourth quartile, 10.7% for patients who required transfusions, 11.8% for patients who had to return to the operating room, 20.5% for patients who had rupture as an indication for surgery, 21.3% for patients who had a myocardial infarction in the post-operative period, 37.6% for patients who required a pre-operative transfusion, 69.7% for patients who were on a ventilator for more than 48 hours, and 99.9% for patients who had all of these seven factors present (Table 3).

**Table 3:** Predicted probability of increased length of hospital stay. OR: Operating room.

Operation time 4^th ^quartile	Post-operative blood transfusions	Return to OR	Ruptured aneurysm	Myocardial infarction	Pre-operative transfusion	Ventilator >48 hours	Probability (%)
+	-	-	-	-	-	-	8.0
-	+	-	-	-	-	-	10.7
-	-	+	-	-	-	-	11.8
-	-	-	+	-	-	-	20.5
-	-	-	-	+	-	-	21.3
-	-	-	-	-	+	-	37.6
-	-	-	-	-	-	+	69.7
+	+	+	+	+	+	+	99.9

## Discussion

This analysis shows that multiple factors play a role in LOS greater than seven days. Preoperative and intraoperative variables include preoperative transfusion, rupture as an indication for surgery, perioperative transfusions, and greater operation time. Postoperative variables include return to the operating room, myocardial infarction, and ventilator dependency duration greater than 48 hours.

Preoperative variables relating to longer LOS in patients have been extensively studied. In a study done by Siracuse et al., the authors identified dependent functional status as the most significant risk factor for protracted LOS in patients undergoing lower extremity bypass for critical limb ischemia [12]. This was also seen in a study by King et al. evaluating risk factors for increased LOS after elective EVAR. The authors noted the importance of identifying preoperative risk factors such as chronic renal insufficiency, recent weight loss, dependent functional status, ASA classification of IV, and dyspnea at rest [13]. These studies emphasize the importance of modifying risk factors in order to optimize surgery but do not evaluate postoperative outcomes as our study has.

Postoperative complication rates have been linked to higher cost of surgical care and have been shown to be more important than preoperative and intraoperative risk factors after major surgery [14]. Additionally, postoperative complications that are more severe can often lead to longer LOS [15]. In our study, postoperative factors that were independently linked to a longer length of stay included return to the operating room, myocardial infarction, and ventilator dependency greater than 48 hours. Patients with postoperative respiratory failure, defined in this study as a failure to wean from a ventilator after 48 hours, had a 50 times greater risk of longer LOS (Table 2), with a predicted probability of almost 70% (Table 3). Patients who returned to operating room and had postoperative myocardial infarction had a three-fold and six-fold increase in risk for longer hospital stay, respectively. This identifies postoperative respiratory failure as an important risk factor for longer LOS and may present as a target for decreasing healthcare costs and shortening hospital stay. Prior studies have emphasized the significance of respiratory failure in surgery outcomes and have demonstrated its association with mortality and overall cost [14, 16-18].

Length of stay is one of many benchmarks that are used to assess healthcare efficiency as well as cost [1-3]. Given what is known about postoperative respiratory failure and our study results demonstrating its role in patients who have undergone EVAR, optimizing patients’ pulmonary status presents as a potential target to improve unnecessarily long LOS and in turn, healthcare costs.

The current study contains some inherent limitations. The NSQIP database is retrospective in nature and therefore has all of the associated limitations of a retrospective study. Outcomes are limited to 30 postoperative days and contain no further follow-up data. Additionally, the database provides no information regarding socioeconomic status or education level. The strength of this study is that NSQIP database has been proven to be reliable and reproducible, and is the most comprehensive surgical database available in the US. Furthermore, the data is collected from multiple institutions throughout the country, thus removing potential geographic biases.

## Conclusions

Multiple factors affect length of hospital stay in patients who have undergone EVAR. Preoperative and intraoperative variables include preoperative transfusion, rupture indication for surgery, perioperative transfusions, and greater operation time. Postoperative variables include return to the operating room, myocardial infarction, and ventilator dependency greater than 48 hours. This study suggests that patients with postoperative respiratory failure after EVAR have a significantly higher risk for longer hospital stays.
